# Retraction: MicroRNA-10a Is Down-Regulated by DNA Methylation and Functions as a Tumor Suppressor in Gastric Cancer Cells

**DOI:** 10.1371/journal.pone.0283120

**Published:** 2023-03-29

**Authors:** The PLOS ONE Editors

The *PLOS ONE* Editors retract this article [1] because it was identified as one of a series of submissions for which we have concerns about peer review, authorship, and similarities across articles. Image similarities were noted between Fig 5B in [1] and Figs 3A and B in [2, 3]. These concerns call into question the validity and provenance of the reported results. We regret that the issues were not addressed prior to the article’s publication.

YM and JY agreed with the retraction. HJ, ZZ, DZ, BW, YY, ML, LD, HY, BG, ZL, FW, WS, CL, JZ, HZ, and JL either did not respond directly or could not be reached.
